# Dopamine Receptor D1 Contributes to Cocaine Epigenetic Reprogramming of Histone Modifications in Male Germ Cells

**DOI:** 10.3389/fcell.2020.00216

**Published:** 2020-04-03

**Authors:** Betina González, Samanta N. Gancedo, Sahira A. Janeir Garazatua, Eduardo Roldán, Alfredo D. Vitullo, Candela R. González

**Affiliations:** ^1^Instituto de Investigaciones Farmacológicas, Universidad de Buenos Aires–Consejo Nacional de Investigaciones Científicas y Técnicas, Buenos Aires, Argentina; ^2^Centro de Estudios Biomédicos Básicos, Aplicados y Desarrollo, Universidad Maimónides, Buenos Aires, Argentina; ^3^Departamento de Biodiversidad y Biología Evolutiva, Museo Nacional de Ciencias Naturales, Madrid, Spain

**Keywords:** cocaine, male germ cells, epigenetics, dopamine receptor 1, histone post-traslational modifications

## Abstract

Paternal environmental perturbations, including cocaine intake, can affect the development and behavior of the offspring through epigenetic inheritance. However, the mechanism by which cocaine alters the male germ cells epigenome is almost unexplored. Here, we report that cocaine-treated male mice showed alterations on specific histone post-translational modifications (PTMs) including increased silent chromatin marks H3K9me3 and H3K27me3 and decreased active enhancer and promoter marks H3K27ac and H3K4me3 in isolated germ cells. Also, cocaine increased H3K9ac and H4K16ac levels, involved in the replacement of histones by protamines that take place at round spermatid stage. Cocaine also altered histones H3/H4 epigenetic enzymes by increasing acetyltransferase KAT8/MOF, deacetylase SIRT1 and methyltransferase KMT1C/G9A, and decreasing deacetylases HDAC1/2 and demethylase KDM1A/LSD1 protein levels. Moreover, a pre-treatment with dopamine receptor 1 (DRD1) antagonist SCH23390 (SCH) blocked cocaine effects on H3K4me3, H3K27me3, and H4K16ac epigenetic marks. Interestingly, treatment with SCH-only was able to modify most of the histone marks tested here, pointing to a dopamine role in controlling histone PTMs in germ cells. Taken together, our data suggest a key role for DRD1 in mediating cocaine-triggered epigenetic modifications related to the silencing of gene transcription and the histone-to-protamine replacement that controls chromatin architecture of maturing sperm cells, and pinpoints a novel role of the dopaminergic system in the regulation of male germ cells reprogramming.

## Introduction

In the last years, there has been special interest in the characterization of epigenetic mechanisms during spermatogenesis that control the reprogramming of the paternal genome, due to the possible trans-generational transmission of acquired traits (Lacal and Ventura, 2018; Galan et al., 2019). Epigenetic reprogramming involves histones post-translational modifications (PTMs), DNA methylation, and changes in small non-coding RNAs that modulate gene expression in response to basal transcriptional programs and environmental signals (Jenkins and Carrell, 2012). Histones PTMs differentially signal chromatin states such as open/transcription-permissive or closed/repressed, as well as regulatory elements in DNA including active enhancers and promoters (Miller and Grant, 2013). The spermatogenesis in particular is characterized by an epigenetic program that enables the multiple chromatin reorganizations and unique transcriptional regulation that are required for proper meiotic divisions and sperm maturation. During spermiogenesis, the histone-to-protamine replacement occurs to facilitate chromatin compaction in the sperm, and histones H3/H4 hyperacetylation is essential for this process (Hazzouri et al., 2000; Steilmann et al., 2011; Shirakata et al., 2014; Bao and Bedford, 2016). Importantly, not all histones are removed from the sperm nucleus; a small percentage (5–15%, depending on the species) is retained at specific loci of key spermatogenesis and embryonic developmental genes (Rajender et al., 2011; Carrell, 2012). It is important to point out that, once paternal DNA compaction has occurred, epigenetic marks may not be altered, creating windows of vulnerability in male germs cells to environmental reprogramming during spermatogenesis (Bale, 2015).

In line with this, recent evidence suggests that cocaine administration in animal models can trigger non-genetic inheritance of addiction traits from father to offspring, including negative birth outcomes, increased rates of anxiety and depression as well as impaired cognition affecting development and behavior (Vassoler et al., 2013; White et al., 2016; Wimmer et al., 2017). This paternal transmission is partly due to the incomplete replacement of histones by protamines. For instance, it has been reported an increased H3K9K14ac2 mark associated with the *Bdnf* promoter in the sperm of cocaine-experienced rats as well as their male offspring (Vassoler et al., 2013). Also, we have recently reported that chronic cocaine treatment increased specific germ cell H3/H4 acetylation (González et al., 2018). Thus, histones PTMs represent epigenetic marks potentially inheritable to offspring (Rajender et al., 2011; Vassoler et al., 2013). However, the mechanism by which cocaine alters male germ cells epigenome has been poorly investigated.

Cocaine intake has been associated with impaired male reproductive function including increased oxidative stress, fibrosis of the seminiferous tubules and germ cell apoptosis that leads to a reduction in sperm production (Bracken et al., 1990; Rodriguez et al., 1992; George et al., 1996; Li et al., 1999; Brown et al., 2006; Fronczak et al., 2012; González et al., 2015). In other tissues, cocaine binds to transporters, receptors, voltage-gated ion channels, and plasma proteins and metabolic enzymes (Heard et al., 2008). Importantly, cocaine inhibits monoamine transporters increasing the synaptic concentration of dopamine, nor-epinephrine and serotonin, and which is responsible for cocaine reinforcing and sympathomimetic effects (Heard et al., 2008). It has been established that the major adverse effects of cocaine are due to increased dopamine binding to dopamine receptor 1 (DRD1) in the mesocorticolimbic system (Anderson and Pierce, 2005; Heard et al., 2008). Concerning the testis, we have previously described that cocaine administration in mice increases tyrosine hydroxylase expression, the rate-limiting enzyme of catecholamine synthesis, and downregulates DRD1 and DRD2, similarly to the mechanism described in the brain (González et al., 2015). Interestingly, we found that the DRD1 receptor was the only one expressed in the spermatogonia nearest the basal lamina of the seminiferous tubules (González et al., 2015). In line with this, early reports have found dopamine located in the wall of seminiferous tubules and interstitial cells (Zieher et al., 1971). Taken together, these findings suggest that the dopamine system could be involved in the epigenetic reprogramming of germ cells.

To date, the mechanisms by which environmental traits can be codified into the male germ cells epigenome and transmitted to the progeny are the focus of intense research. Here, we evaluated the effects of chronic cocaine treatment in adult male mice, and participation of DRD1, on specific histone modifications and epigenetic enzymes, and involved in the structural and dynamic changes of chromatin in isolated male germ cells.

## Materials and Methods

### Animals

Male C57BL/6 mice (10–12 weeks old) from the School of Exact and Natural Sciences of the University of Buenos Aires (UBA) were housed in a light- and temperature-controlled room. Principles of animal care were followed in accordance with “Guidelines for the Care and Use of Mammals in Neuroscience and Behavioral Research” (National Research Council (US) Committee on Guidelines for the Use of Animals in Neuroscience and Behavioral Research, 2003) and approved by IACUC Committee of the Faculty of Pharmacy and Biochemistry, Universidad de Buenos Aires (Protocol Number: EXP-FYB N° 52867/2019 RES(D) N° 2019-3534).

### Pharmacological Treatment

Mice were treated with cocaine (Sigma-Aldrich, St. Louis, MO, United States) or vehicle (sterile 0.9% saline), in an intermittent *binge* protocol: 3 i.p. injections, 1 h apart, one day on/off for 13 days (González et al., 2015, 2018). To evaluate the involvement of DRD1 in the deleterious action of cocaine, DRD1 antagonist SCH23390 (TOCRIS bioscience, Ellisville, MO, United States) was injected 15 min before each cocaine or vehicle injection (González et al., 2016). Animals were assigned to four different groups: COC (3 × saline s.c + 3 × cocaine 10 mg/kg i.p), SCH (3 × SCH23390 0.5 mg/kg s.c + 3 × saline i.p), SCH-COC (3 × SCH23390 0.5 mg/kg s.c + 3 × cocaine 10 mg/kg i.p), and VEH (3 × saline s.c + 3 × saline i.p). Mice were euthanized 24 h after the last binge on day 14 and testes removed for isolation of germ cells.

### Germ Cells Isolation

Germ cells were isolated from the testes of the four experimental groups as was previously described (González et al., 2018). The right and left testes of each animal were decapsulated and digested with type I collagenase (0.23 mg/ml, Sigma) in phosphate-buffered saline (PBS) with 0.1% bovine serum albumin for 10 min at 34°C in a shaking water bath. Collagenase activity was stopped by adding cold PBS and the seminiferous tubules were allowed to settle and washed three times with PBS. Then, the seminiferous tubules were mechanically dispersed and the supernatants were filtered (cell strainer, 41 μm) and centrifuged at 150 g for 15 min. Finally, PBS was removed and the cells were kept at −80°C for molecular studies.

### Western Blot

Western blot analyses were conducted as previously described (González et al., 2018). Briefly, homogenates were prepared in a solution containing 50 mM Tris–HCl pH 7.5, 150 mM NaCl, 0.1% Triton X100, 0.5% sodium deoxycholate, 0.1% SDS, 1 mM PMSF, 5 μg/ml leupeptin, and 5 μg/ml aprotinin. After removal of cell debris by centrifugation, the protein concentration of the cell lysate was determined. The homogenates were combined with loading buffer containing 4% SDS, 20% glycerol, 10% β-mercaptoethanol, 125 mM Tris, (pH 6.8), and boiled at 100°C for 5 min. Protein samples (15–50 μg) were separated by 10–12% SDS-PAGE, and the proteins transferred to a PVDF membrane. Blots were incubated with the following primary antibodies: anti-DRD1 (1:50, sc33660), anti-HDAC2 (1:1000, sc-7899), anti-G9a (1:250, sc515726), anti-Tip60 (1:250, sc166323), anti-MOF (1:250, sc81163), anti-LSD1 (1:250, sc271720), and anti-SIRT1 (1:500, sc74465) from Santa Cruz Biotechnology Inc., United States; anti-H3K4me3 (1:1500, ab1012), anti-H3K27me3 (1:250, ab192985), anti-H4K16ac (1:250, ab109463), anti-H3K9me3 (1:1000, ab8898), and anti-H3K27ac (1:1000, ab4729) from Abcam, United Kingdom; anti-HDAC1 (1:1000, 05-100-I) from Millipore, PAIS; anti-H3K9ac (1:500, #9649) from Cell Signaling Technology, United States; and anti-Actin (1:5000, A5441) and anti-a-tubulin (1:10000, T9026) were from Sigma, United States. Immune complexes were detected with anti-rabbit or anti-mouse secondary antibodies and chemiluminescence reagents (Amersham, United States), and bands were visualized with the image reader ImageQuant 350 (GE Healthcare). The resulting images were quantified with ImageJ (NIH) software. Original blots shown in this study are available in Supplementary Material.

### Real Time PCR

RT-PCR was conducted as previously described (González et al., 2015, 2018). Briefly, total germ cells RNA was extracted with TRIzol (Invitrogen, Carlsbad, CA, United States) according to the manufacturer’s instructions. Total RNA (1 μg) was treated with DNAseI (Invitrogen, Carlsbad, CA, United States) and used for reverse transcription in a 20 μl final volume containing M-MLV reverse transcriptase (200 U/μl) (Promega, Madison, WI, United States), and random hexamer primers (Biodynamics, Milwaukee, WI, United States). Reverse transcribed cDNA was employed for quantitative PCR using SYBR Green PCR Master Mix and specific primers in a Stratagene MPX500 cycler (Stratagene, San Diego, United States). Primers sequences for DRD1 and GAPDH are published in González et al. (2015). Data from the reaction were collected and analyzed by the complementary computer software (MxPro3005P v4.10 Build 389, Schema 85). Relative quantitation of gene expression was calculated using standard curves and normalized to GAPDH in each sample.

### Statistical Analysis

Statistics were performed using one-way ANOVA followed by Bonferroni *post hoc* test. Data were transformed when required. For data that did not comply with parametric test assumptions, Kruskal–Wallis ANOVA on ranks followed by paired comparisons was applied. InfoStat 2010 software^1^ was used for statistical analysis. Differences were considered significant if *p* < 0.05.

## Results

### Cocaine Elicits DRD1 Downregulation in Germ Cells via a DRD1-Dependent Mechanism

**FIGURE 1:**
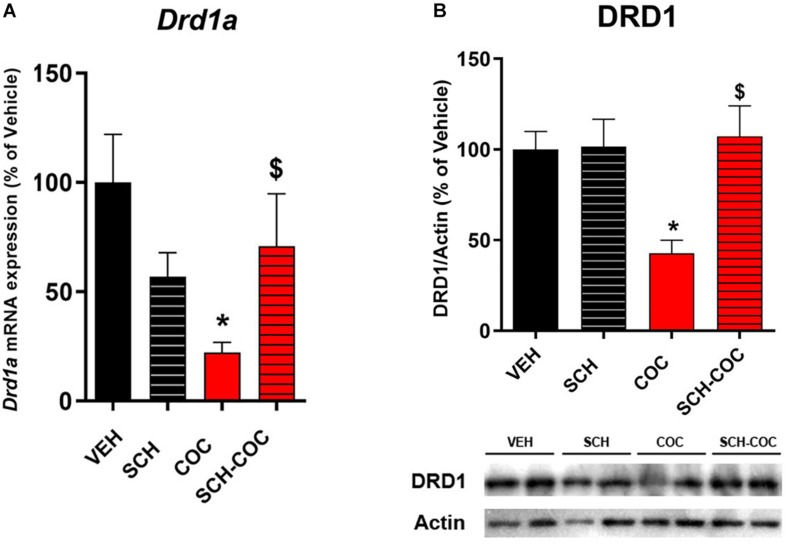
Effect of cocaine (COC), SCH23390 (SCH), and SCH 15 min before COC (SCH-COC) treatments on DRD1 mRNA and protein expression in male germ cells. **(A)** DRD1 mRNA expression (RT-PCR), Kruskal–Wallis-paired comparisons *H* = 12.86, *p* = 0.005. **(B)** DRD1 protein levels (western blot), ANOVA-Bonferroni *F*_(__3_,_23__)_ = 4.49, *p* = 0.014. Values indicate mean ± SEM (*n* = 5–7). *p < 0.05 different from VEH, ^$^*p* < 0.05 different from COC.

We have previously reported that male mice spermatogonia express DRD1, and that cocaine administration affects the testicular dopaminergic system decreasing DRD1 mRNA (González et al., 2015). Therefore, we evaluated the effect of cocaine (COC), DRD1 antagonist SCH23390 (SCH) receptor, and SCH administered 15 min before COC (SCH-COC) on DRD1 expression levels in isolated mouse germ cells. Both DRD1 mRNA (Figure 1A) and protein (Figure 1B) expression were significantly reduced in germ cells from cocaine-treated mice compared to vehicle (VEH). Combined SCH-COC treatment was able to revert the effect of cocaine on DRD1 mRNA and protein levels (Figure 1). No differences in mRNA and protein expression of DRD1 were detected in SCH group compared to VEH under these experimental conditions.

### Cocaine Elicits Epigenetic Reprogramming of Histone PTMs in Male Germ Cells: Role of DRD1

**FIGURE 2:**
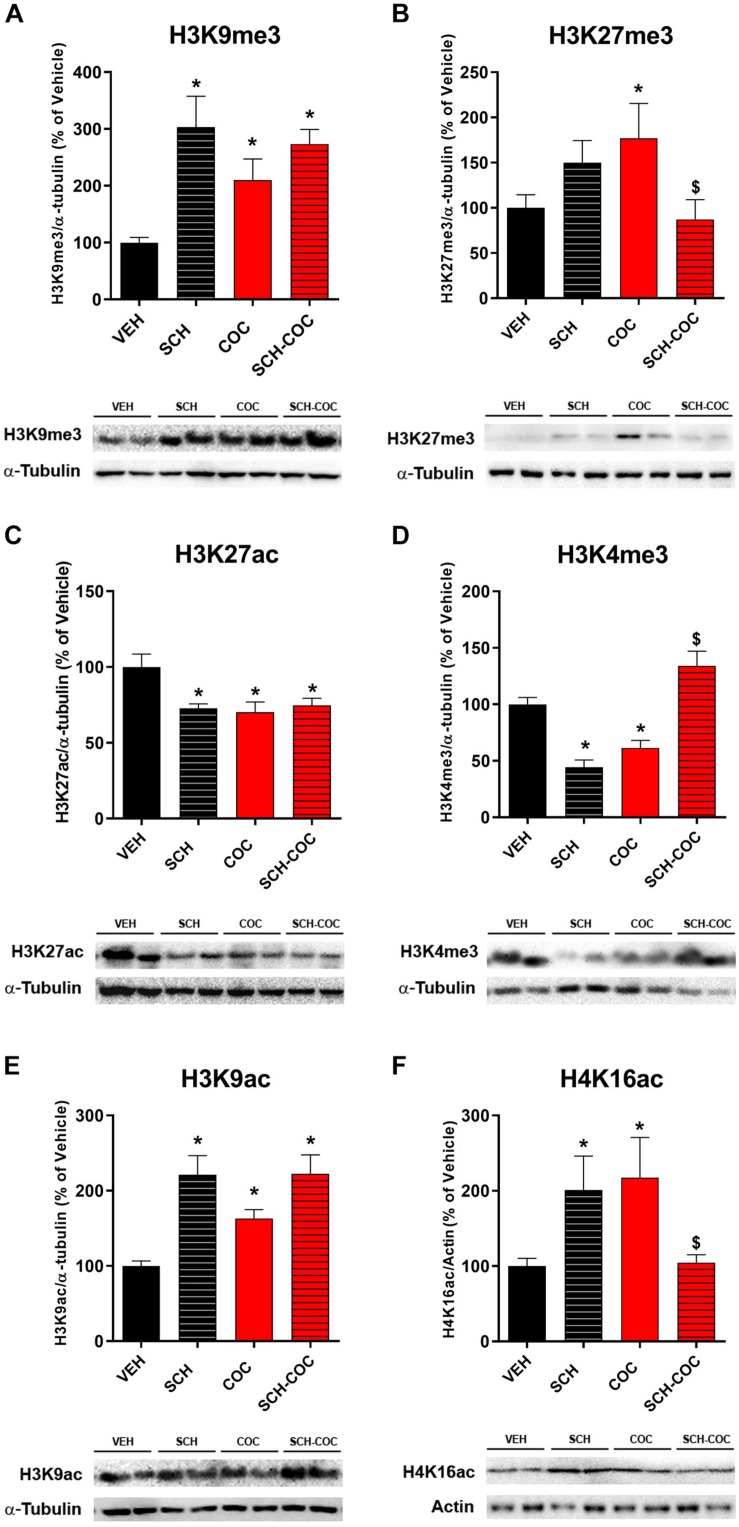
Effect of cocaine (COC), SCH23390 (SCH), and SCH 15 min before COC (SCH-COC) treatments on H3 and H4 post-traslational modifications expression in male germ cells. Protein expression levels (western blot) of **(A)** H3K9me3, ANOVA-Bonferroni *F*_(__3,20__)_ = 6.87, *p* = 0.004. **(B)** H3K27me3, Kruskal–Wallis-paired comparisons *H* = 7.93, *p* = 0.047. **(C)** H3K27ac, ANOVA-Bonferroni *F*_(__3,20__)_ = 8.86, *p* = 0.0009. **(D)** H3K4me3, ANOVA-Bonferroni *F*_(__3,23__)_ = 23.84, *p* < 0.0001. **(E)** H3K9ac, Kruskal–Wallis-paired comparisons *H* = 13.5, *p* = 0.004. **(F)** H4K16ac, Kruskal–Wallis-paired comparisons *H* = 12.6, *p* = 0.005. Values indicate mean ± SEM (*n* = 5–7). *p < 0.05 different from VEH, _$_*p* < 0.05 different from COC.

We evaluated the effect of COC, SCH, and SCH-COC treatments on protein expression levels of specific H3/H4 PTMs related to the epigenetic regulation of gene expression and chromatin remodeling during spermatogenesis: (i) H3K9me3 and H3K27me3 as silent chromatin marks, (ii) H3K27ac and H3K4me3 as active enhancer and promoter marks, and (iii) H3K9ac and H4K16ac as marks of open chromatin states and the replacement of histones by protamines. We found a significant increase in H3K9me3, H3K27me3, H3K9ac, and H4K16ac protein levels in isolated germ cells of COC group compared to VEH (Figure 2). Pre-treatment with SCH counteracted cocaine-increased protein levels of H4K16ac and H3K27me3 (Figure 2). On the other hand, we observed a decrease in H3K27ac and H3K4me3 in isolated germ cells of COC group compared to VEH (Figure 2). Pre-treatment with SCH was able to re-establish protein levels of H3K4me3 (Figure 2).

### Cocaine Affects the Expression of Epigenetic Enzymes: Role of DRD1

**FIGURE 3:**
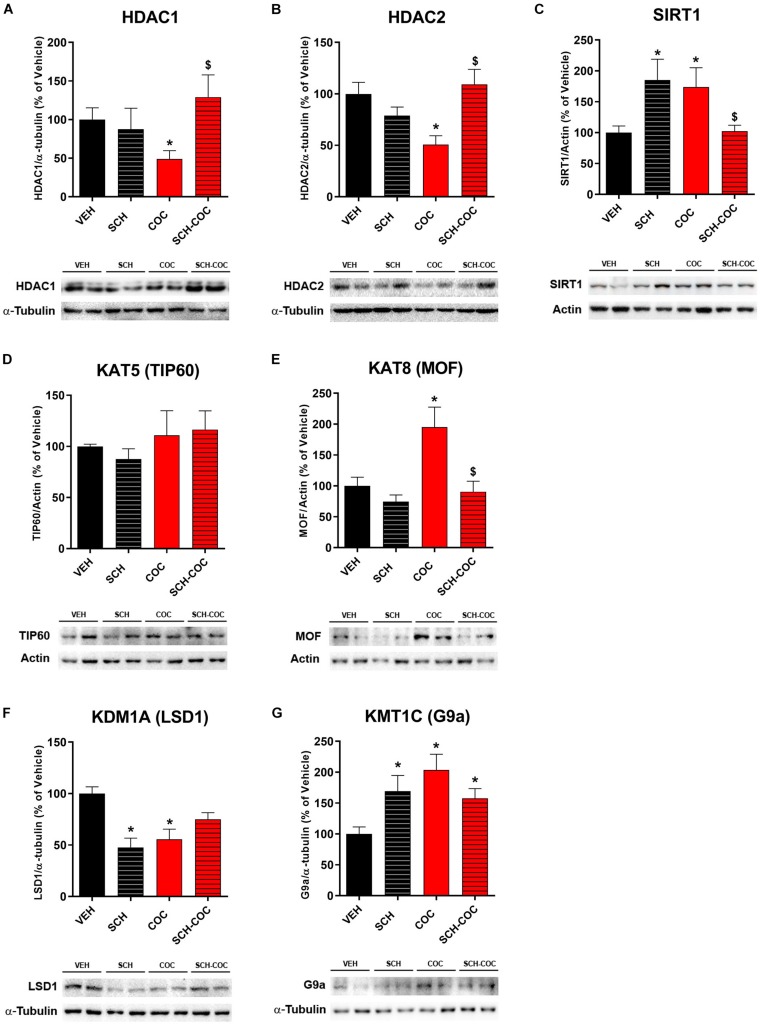
Effect of cocaine (COC), SCH23390 (SCH), and SCH 15 min before COC (SCH-COC) treatments on histone modifying enzymes expression in male germ cells. Protein expression levels (western blot) of **(A)** HDAC1, Kruskal–Wallis-paired comparisons *H* = 7.9, *p* = 0.048. **(B)** HDAC2, ANOVA-Bonferroni *F*_(__3_,_24__)_ = 6.32, *p* = 0.003. **(C)** SIRT1, Kruskal–Wallis-paired comparisons *H* = 10.2, *p* = 0.016. **(D)** TIP60, ANOVA-Bonferroni *F*_(__3_,_20__)_ = 1, *p* > 0.05. **(E)** MOF, ANOVA-Bonferroni *F*_(__3_,_24__)_ = 6.8, *p* = 0.002. **(F)** LSD1, ANOVA-Bonferroni *F*_(__3_,_20__)_ = 9.5, *p* = 0.0009. **(G)** G9a, Kruskal–Wallis-paired comparisons *H* = 11.3, *p* = 0.01. *p < 0.05 different from VEH, ^$^*p* < 0.05 different from COC.

We evaluated protein expression levels of histone modifying enzymes acetyltransferases/deacetylases (KATs/HDACs) and methyltransferases/demethylases (KMTs/KDMs) in isolated germs cells from all groups. We found decreased protein levels of class I deacetylases HDAC1 and HDAC2 and increased class III deacetylase SIRT1 in COC group compared to VEH, which reverted under pre-treatment with SCH (Figure 3). SCH-only treatment was also able to increase SIRT1 (Figure 3). Protein levels of acetyltransferase KAT8/MOF, which catalyzes the specific acetylation of H4K16, increased in COC group compared to VEH and reverted under pre-treatment with SCH (Figure 3). No differences in KAT5/TIP60 protein levels were detected between groups under these experimental conditions (Figure 3). We also found that KDM1A/LSD1, which can demethylate both H3K4me and H3K9me, decreased both in COC and SCH groups compared to VEH, whereas SCH-COC was neither different from VEH nor COC groups. Protein levels of KMT1C/G9A, which catalyzes H3K9 methylation, increased in SCH, COC, and SCH-COC groups compared to VEH (Figure 3).

## Discussion

The once controversial idea that parental lifestyle can shape the physiology and behavior of their offspring via epigenetic inheritance has become a vibrant area of research. Accumulating data has shown that male germ cells are epigenetically modified at various time points during spermatogenesis to condense and protect paternal DNA, and also to provide epigenetic information for future embryo development. Here, we report that cocaine, through both DRD1-dependent and independent mechanisms, altered specific histones PTMs and epigenetic modifying enzymes related to the control of gene transcription and to the histone-to-protamine replacement, suggesting a novel role for the dopaminergic system in the regulation of germ cells reprogramming.

It has been found that H3 retention sites in normal sperm are highly conserved, and specific PTMs alterations were linked to epigenetic transgenerational transmission of environmental toxicants exposure traits (Ben Maamar et al., 2018). Retained H3 PTMs in the sperm epigenome were located at key genes that control the spermatogenesis, showing a so-called “spermatogenic memory,” as well as at developmental genes that will take part in the future embryonic program (Carrell and Hammoud, 2010). The active transcription mark H3K4me3 was highly detected in the sperm nucleosome fraction and enriched at gene clusters of developmental genes, non-coding RNAs and spermatogenesis-related genes (Erkek et al., 2013). Also, sperm-retained H3K27ac was found enriched at super-enhancers that are active in adult tissues, suggesting that cis-regulatory elements critical for adult cell differentiation are already specified in sperm (Jung et al., 2017). On the other hand, H3K9me3 was found retained at satellite repeats in mouse sperm, whereas H3K27me3 was found enriched in promoters of developmental genes that are repressed in the early pre-implantation stages of embryogenesis (Hammoud et al., 2009; Brykczynska et al., 2010; Carrell, 2012; Erkek et al., 2013). Here, we found that cocaine treatment increased silent chromatin marks H3K9me3/H3K27me3 and decreased active enhancer and promoter marks H3K27ac/H3K4me3 in mouse germ cells. It has been shown that DNA methylation induces H3K27me3 deposition at specific gene promoters (Hammoud et al., 2009; King et al., 2016), and we have previously found that cocaine increased 5-mC levels in DNA from isolated germ cells and sperm (González et al., 2018). Interestingly, blockade of DRD1 was only able to revert cocaine-induced effects on the functionally opposite histone marks H3K4me3 and H3K27me3. Sperm retained nucleosomes often contain H3K4me3/H3K27me3 bivalent marking, characteristic of gene preactivation termed “poising” (Hammoud et al., 2014), and localize at the promoters of hundreds of developmental genes, including Hox-, Fox-, Sox-, and Gata-families (Hammoud et al., 2009; Brykczynska et al., 2010). Therefore, our data suggest that cocaine, through DRD1 activation, may cause H3K4me3/H3K27me3 imbalance potentially affecting the embryonic developmental program. In line with this, we found that cocaine treatment increased KMT1C/G9A and decreased KDM1A/LSD1 enzymes. KMT1C/G9A is a key mediator of the epigenetic effects of cocaine in the mesolimbic system (Maze et al., 2010; Anderson et al., 2019) and has been described as a crucial epigenetic marker of heterochromatin formation during meiosis (Tachibana et al., 2007). KDM1A/LSD1 participates in the demethylation of H3K4/K9 and is required for spermatogonial differentiation and germ cell survival in mice (Myrick et al., 2017). Also, it has been found that many bivalent genes have increased H3K4me3 and decreased H3K27me3 levels and are occupied by KDM1A/LSD1 to maintain low levels of H3K4me2 that often co-localized with H3K4me3 (Adamo et al., 2011; Whyte et al., 2012). Additionally, KDM1A/LSD1 inactivation results in increased global H3K27me3 leading to suppression of gene expression (Leis et al., 2012). Interestingly, KDM1A/LSD1 was found in the same transcriptional repressor complex with HDAC1/2 (Kelly et al., 2018), which were also downregulated in germ cells after cocaine treatment (González et al., 2018), and its expression tightly correlated with H3K4me3 levels in male germ cells (Godmann et al., 2007). Altogether, these data suggest that cocaine promotes alterations in KDM/KMT enzymes that would trigger altered methylation patterns of H3 lysine residues associated with the silencing of genetic transcription in mouse germ cells.

During spermatogenesis, specific histones PTMs work together to facilitate genome re-organization and packaging of the sperm nucleus. Hyperacetylation of H3K9 and H4K16 triggers the histone-to-protamine replacement, which takes place at stage 8–12 round spermatids (Hazzouri et al., 2000; Steilmann et al., 2011; Shirakata et al., 2014). Here, we found increased H3K9ac and H4K16ac in mouse germ cells after cocaine treatment. We also found altered levels of the H4K16-specific acetyltransferase KAT8/MOF and deacetylases HDAC1/2 and SIRT1, which were all found to participate in the histone-to-protamine transition at round spermatid stage (Fenic et al., 2004; Bell et al., 2014; Jiang et al., 2018). In addition, we found that SCH pre-treatment was able to revert cocaine-induced effects on H4K16ac as well as HDAC1/2, SIRT1, and KAT8/MOF. These data suggest that cocaine, via DRD1 regulation, has a key role in modulating the acetylation status of male epigenome most likely interfering with histone eviction and chromatin reassembly.

The data presented here showed that DRD1 blockade is able to re-establish the levels of some epigenetic marks altered by cocaine. Also, SCH-only treatment was able to modify most of the epigenetic histone PTMs as well as SIRT1, KDM1A/LSD1, and KMT1C/G9A, further supporting a novel role of dopamine controlling epigenetic marks during spermatogenesis. Noteworthy, H4K16ac, H3K4me3, H3K27me3, and SIRT1 showed that SCH-only treatment behaved like cocaine, but returned to control values in the combined SCH-COC group. This type of response is typical of the so-called “inverted U-shaped” effect of DRD1, extensively studied in brain cortical cells, where both low (as in SCH) and high (as in COC) dopamine concentrations impact DRD1 signaling causing similar detrimental effects in cells function and cognition (Williams and Castner, 2006; González et al., 2016). Here, cocaine may have increased local dopamine production by TH-expressing neuron-like-cells and meiotic germ cells (Frungieri et al., 2000; González et al., 2015), as well as plasmatic dopamine through sympathetic nerves and/or adrenal medulla release (Rubí and Maechler, 2010). Also, the DRD1-mediated effects found in germ cells could have been triggered by autocrine-paracrine effects of DRD1-expressing spermatogonia (González et al., 2015), by interstitial-tubular effects of Leydig cells expressing DRD1 (González et al., 2015), and also by endocrine hypothalamic-pituitary factors under the control of the central tubero-infundibular dopamine circuit such as prolactin (Rubí and Maechler, 2010). Our data point to DRD1 involvement in germ cell epigenetic homeostasis, but also, that these cocaine-reprogramming effects in germ cells are potentially reversible. This type of evidence becomes of great importance due to the existence of several therapeutic drugs that affect the dopaminergic system and cause male infertility. For instance, it was found that dopamine antagonists and antidepressants such as reserpine (Yamauchi et al., 2000), dopamine agonist bromocriptine (Richardson et al., 1984), and antihypertensive drug methyldopa (Chapin and Williams, 1989) cause testicular atrophy. Thus, epigenetic changes induced by dopamine imbalance in male germ cells could be reversible if the environmental conditions return to normal.

## Conclusion

In conclusion, our findings strongly suggest that cocaine can induce an epigenetic reprogramming of male germ cells through changes in epigenetic enzymes and histones specific PTMs which could trigger silencing of genetic expression and, moreover, alter the histone-to-protamine replacement event necessary to chromatin reorganization and DNA compaction. Although this is a preliminary study performed in samples containing all the germ cells populations, we show novel evidence that pinpoint a key role for DRD1 in mediating specific epigenetic modifications induced by cocaine in mouse germ cells. Further studies in specific cell stages of spermatogenesis obtained by cell sorting will be performed in order to expand to the knowledge about the mechanisms by which environmental effects such as addictive stimulants consumption can be codified in the paternal epigenome and transmitted across generations.

## Data Availability Statement

The datasets generated for this study are available on request to the corresponding author.

## Ethics Statement

The animal study was reviewed and approved by Principles of animal care were followed in accordance with “Guidelines for the Care and Use of Mammals in Neuroscience and Behavioral Research” (National Research Council (US) Committee on Guidelines for the Use of Animals in Neuroscience and Behavioral Research, 2003) and approved by IACUC Committee of the Faculty of Pharmacy and Biochemistry, Universidad de Buenos Aires (Protocol Number: EXP-FYB N° 52867/2019 RES(D) N° 2019-3534).

## Author Contributions

CG and BG contributed to conception and design of the study. CG, BG, SG, and SJ contributed to the methodology. BG performed the statistical analysis. CG and BG wrote the first draft of the manuscript. AV and ER contributed to the writing and editing of the manuscript. All authors read and approved the submitted version.

## Conflict of Interest

The authors declare that the research was conducted in the absence of any commercial or financial relationships that could be construed as a potential conflict of interest.

## Footnotes

1 www.infostat.com.ar
