# Supplementary material for: Dopamine Receptor D1 Contributes to Cocaine Epigenetic Reprogramming of Histone Modifications in Male Germ Cells
Source: Front Cell Dev Biol. 2020 Apr 3;8:216. doi: 10.3389/fcell.2020.00216 (PMC7146055; doi:10.3389/fcell.2020.00216)
Supplement: Supplementary file 1 [file Data_Sheet_1.PDF]

Supplementary Material

DOPAMINE RECEPTOR D1 CONTRIBUTES TO COCAINE EPIGENETIC REPROGRAMMING OF HISTONE MODIFICATIONS IN MALE GERM CELLS

Betina González, Samanta N. Gancedo, Sahira A. Janeir Garazatua, Eduardo Roldán, Alfredo D. Vitullo, Candela R. González

The following images represent the raw data obtained for each western blot experiment showed in the manuscript. The red rectangles show the portion of the image that is shown in the manuscript figures.

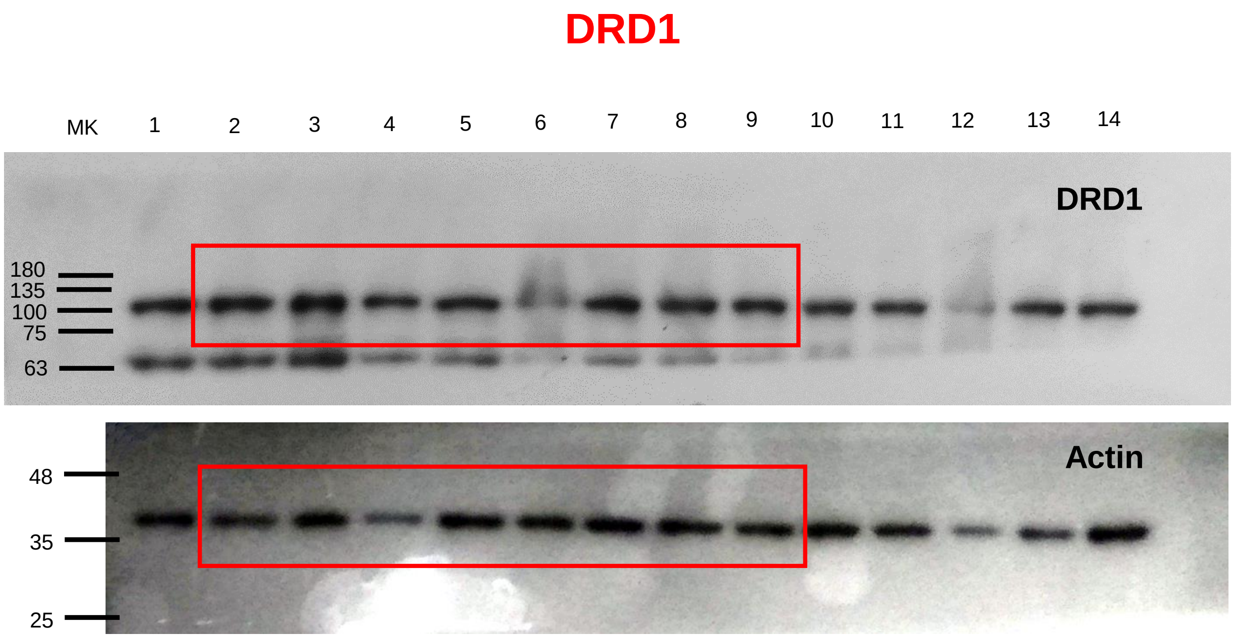

Lanes: MK) molecular weight marker (kDa), 1) brain frontal cortex, 2) veh1, 3) veh2, 4) sch1, 5) sch2, 6) coc1, 7) coc2, 8) sch-coc1, 9) sch-coc2, 10) veh3, 12) sch3, 13) coc3, 14) sch-coc3, 15) naive

H3K9me3

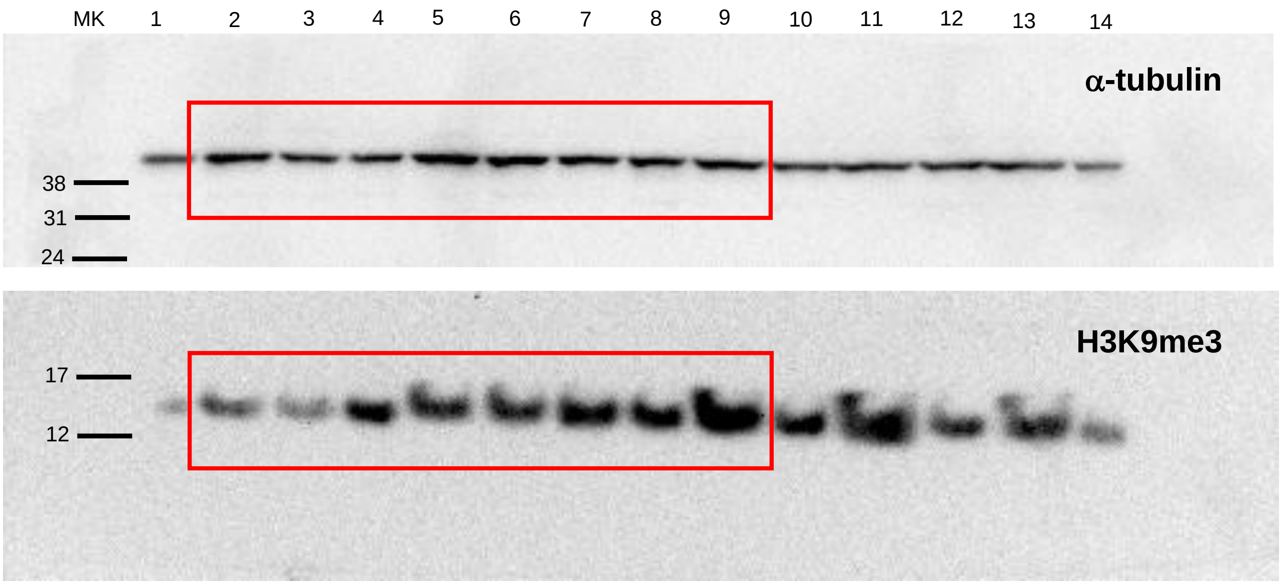

Lanes: MK) molecular weight marker (kDa), 1) naive, 2) veh1, 3) veh2, 4) sch1, 5) sch2, 6) coc1, 7) coc2, 8) sch-coc1, 9) sch-coc2, 10) coc3, 11) sch3, 12) coc4, 13) sch-coc4, 14) veh3

H3K27me3

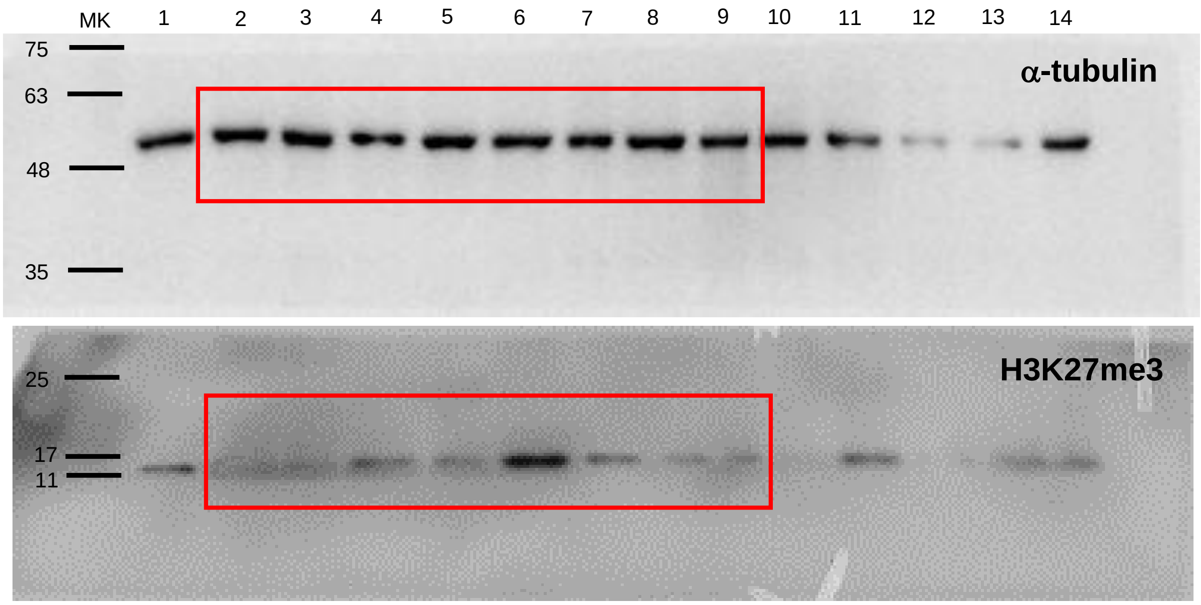

Lanes: MK) molecular weight marker (kDa), 1) brain frontal cortex, 2) veh1, 3) veh2, 4) sch1, 5) sch2, 6) coc1, 7) coc2, 8) sch-coc1, 9) sch-coc2, 10) veh3, 11) sch3, 12) coc3, 13) sch-coc3, 14) Naive

H3K27ac

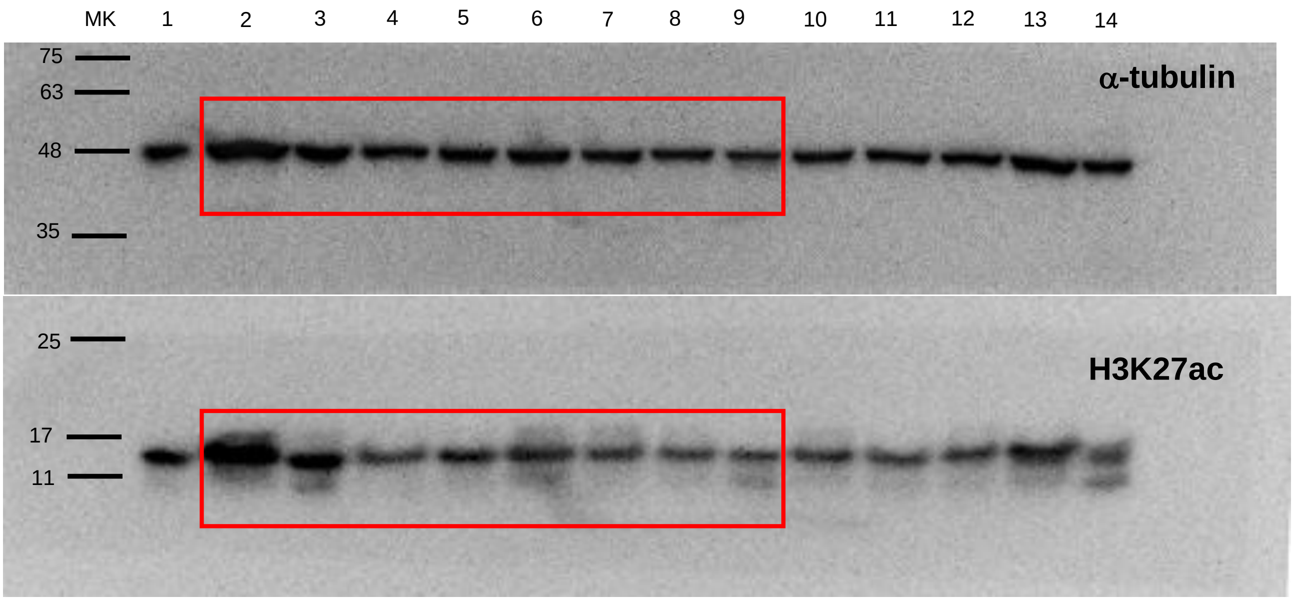

Lanes: MK) molecular weight marker (kDa), 1) naive, 2) veh1, 3) veh2, 4) sch1, 5) sch2, 6) coc1, 7) coc2, 8) sch-coc1, 9) sch-coc2, 10) coc3, 11) sch3, 12) coc4, 13) sch-coc4, 14) veh3

H3K4me3

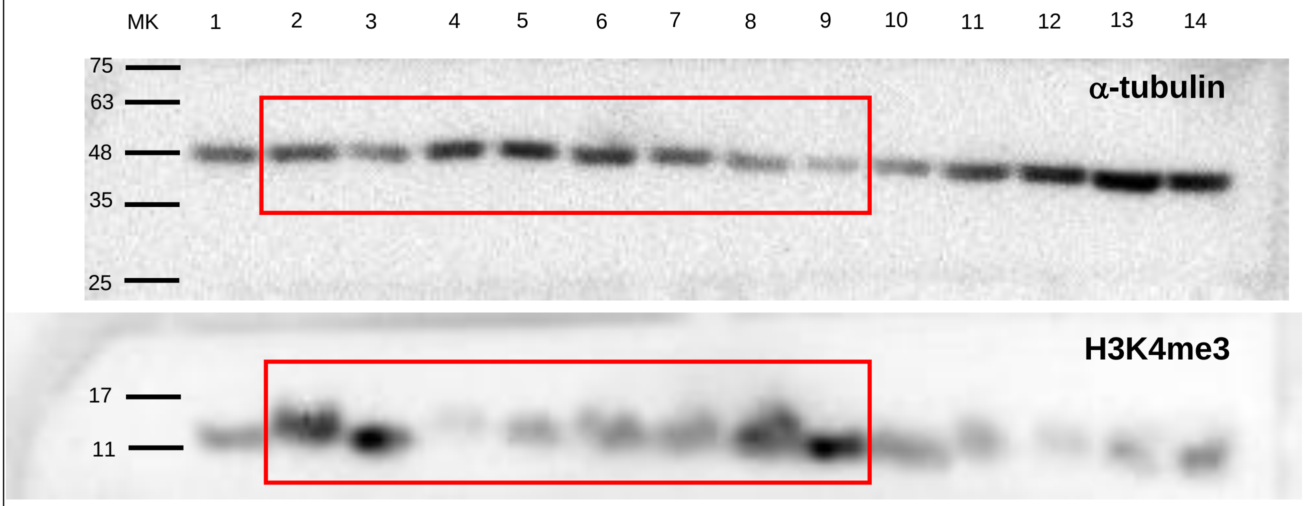

Lanes: MK) molecular weight marker (kDa), 1) naive, 2) veh1, 3) veh2, 4) sch1, 5) sch2, 6) coc1, 7) coc2, 8) sch-coc1, 9) sch-coc2, 10) coc3, 11) sch3, 12) coc4, 13) sch-coc4, 14) veh3

H3K9ac

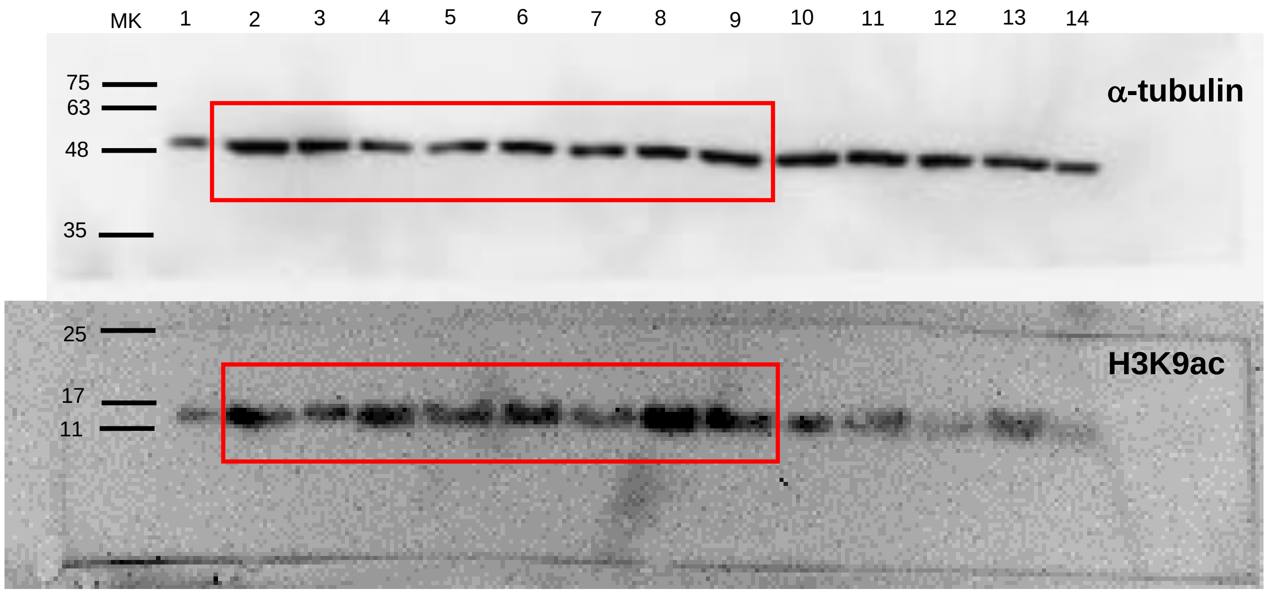

Lanes: MK) molecular weight marker (kDa), 1) naive, 2) veh1, 3) veh2, 4) sch1, 5) sch2, 6) coc1, 7) coc2, 8) sch-coc1, 9) sch-coc2, 10) coc3, 11) sch3, 12) coc4, 13) sch-coc4, 14) veh3

H4K16ac

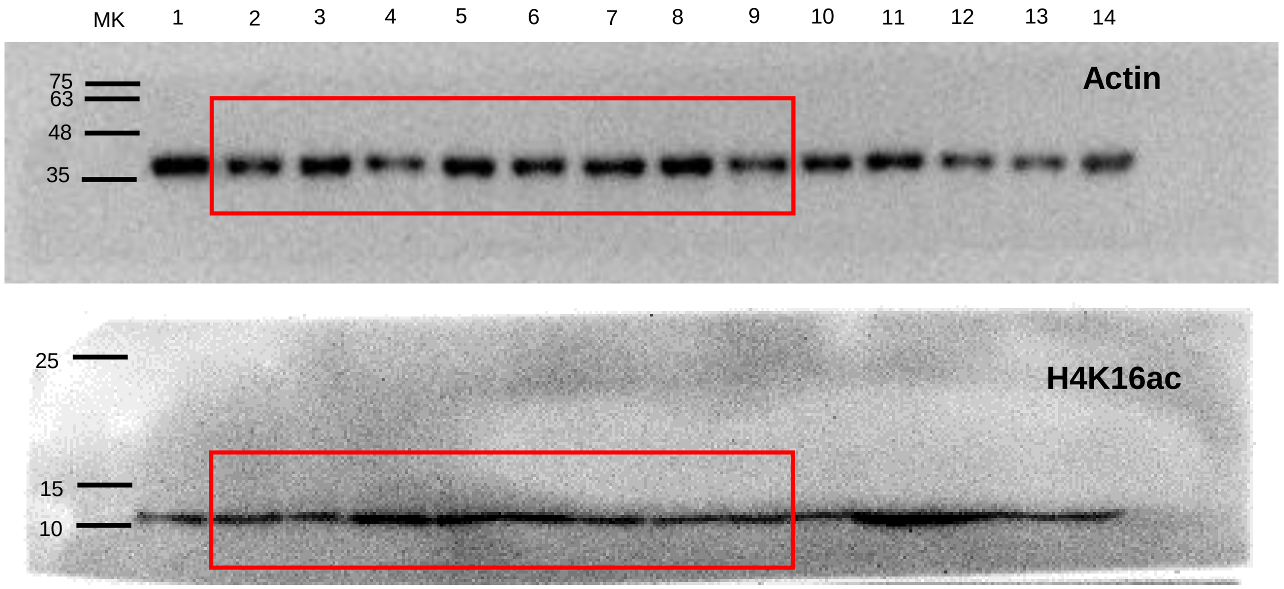

Lanes: MK) molecular weight marker (kDa), 1) brain frontal cortex, 2) veh1, 3) veh2, 4) sch1, 5) sch2, 6) coc1, 7) coc2, 8) sch-coc1, 9) sch-coc2, 10) veh3, 11) sch3, 12) coc3, 13) sch-coc3, 14) naive

HDAC1

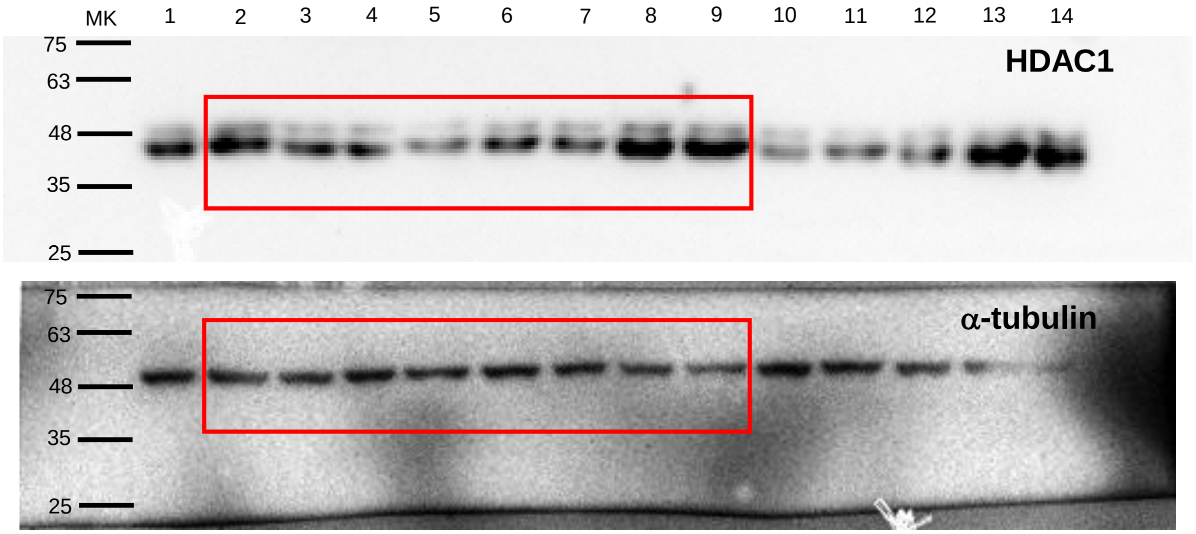

Lanes: MK) molecular weight marker (kDa), 1) naive, 2) veh1, 3) veh2, 4) sch1, 5) sch2, 6) coc1, 7) coc2, 8) sch-coc1, 9) sch-coc2, 10) coc3, 11) sch3, 12) coc4, 13) sch-coc4, 14) veh3

HDAC2

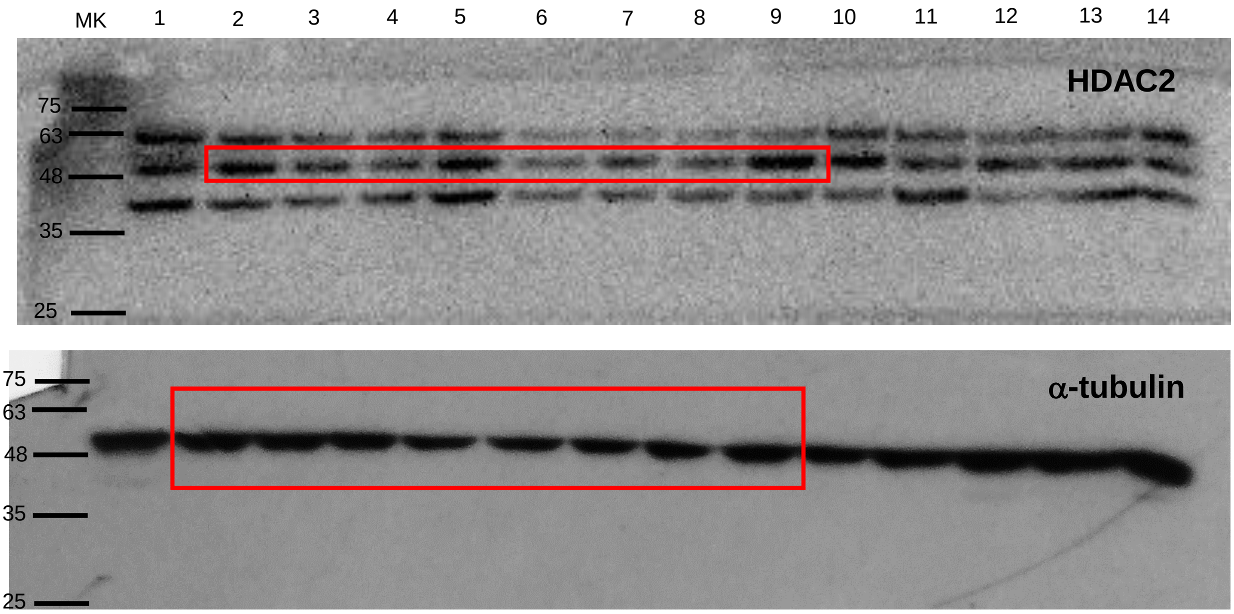

Lanes: MK) molecular weight marker (kDa), 1) brain frontal cortex, 2) veh1, 3) veh2, 4) sch1, 5) sch2, 6) coc1, 7) coc2, 8) sch-coc1, 9) sch-coc2, 10) veh3, 11) sch3, 12) coc3, 13) sch-coc3, 14) naive

SIRT1

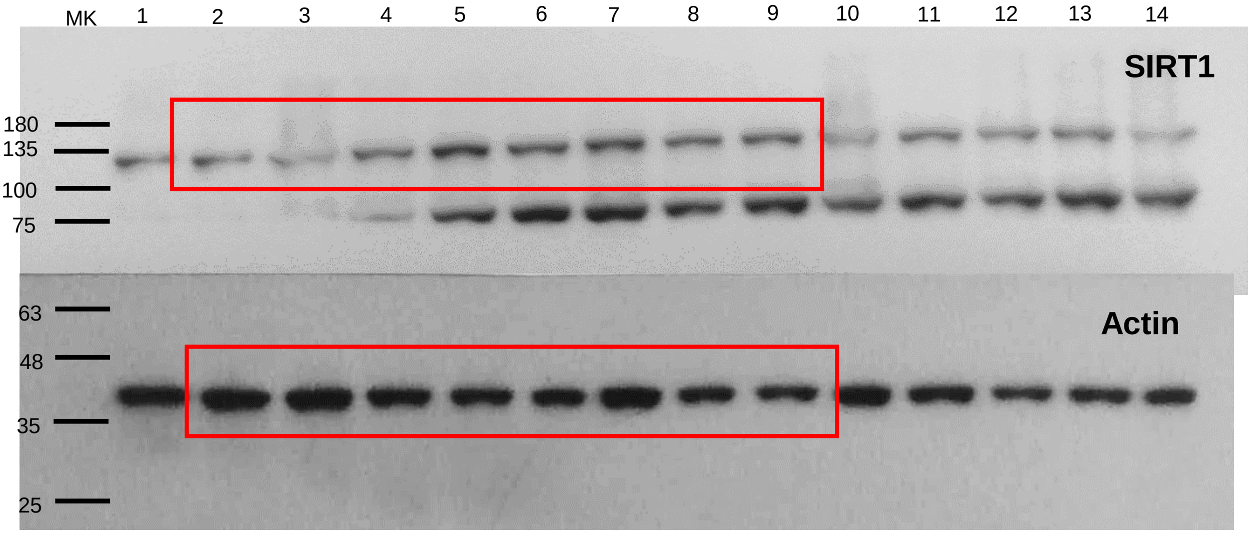

Lanes: MK) molecular weight marker (kDa), 1) naive, 2) veh1, 3) veh2, 4) sch1, 5) sch2, 6) coc1, 7) coc2, 8) sch-coc1, 9) sch-coc2, 10) coc3, 11) sch3, 12) coc4, 13) sch-coc4, 14) veh3

TIP60

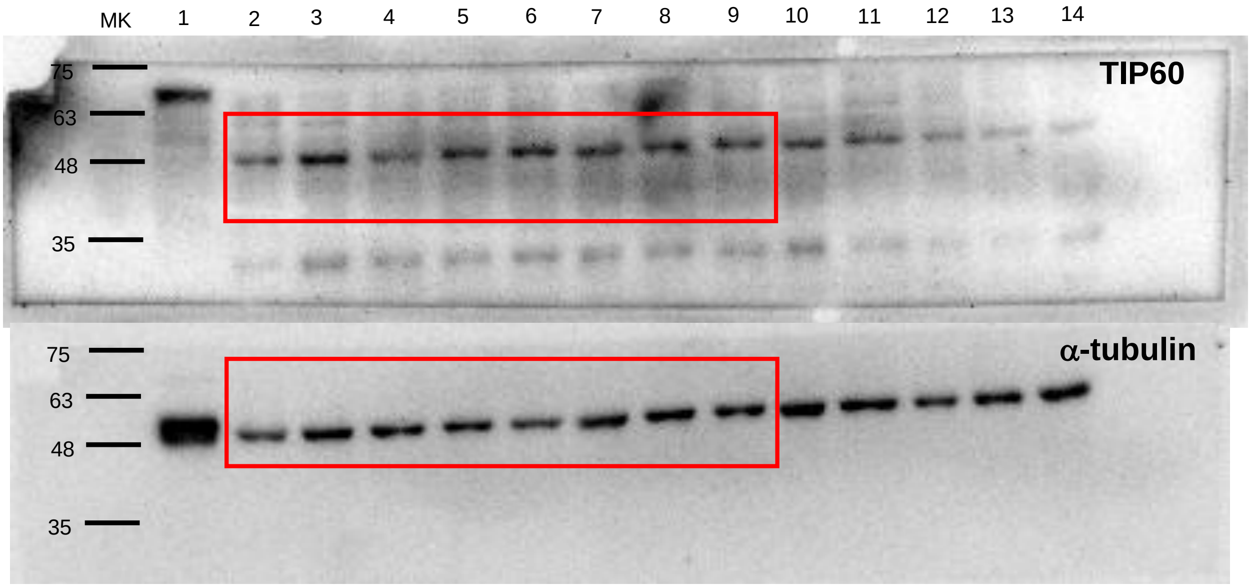

Lanes: MK) molecular weight marker (kDa), 1) brain frontal cortex, 2) veh1, 3) veh2, 4) sch1, 5) sch2, 6) coc1, 7) coc2, 8) sch-coc1, 9) sch-coc2, 10) veh3, 11) sch3, 12) coc3, 13) sch-coc3, 14) naive

MOF

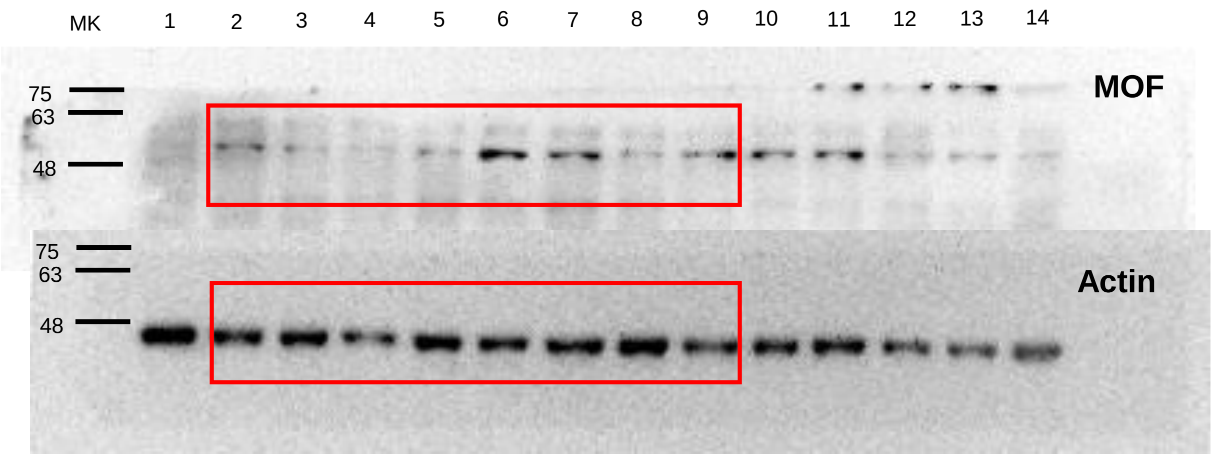

Lanes: MK) molecular weight marker (kDa), 1) naive, 2) veh1, 3) veh2, 4) sch1, 5) sch2, 6) coc1, 7) coc2, 8) sch-coc1, 9) sch-coc2, 10) coc3, 11) sch3, 12) coc4, 13) sch-coc4, 14) veh3

KDM1A/LSD1

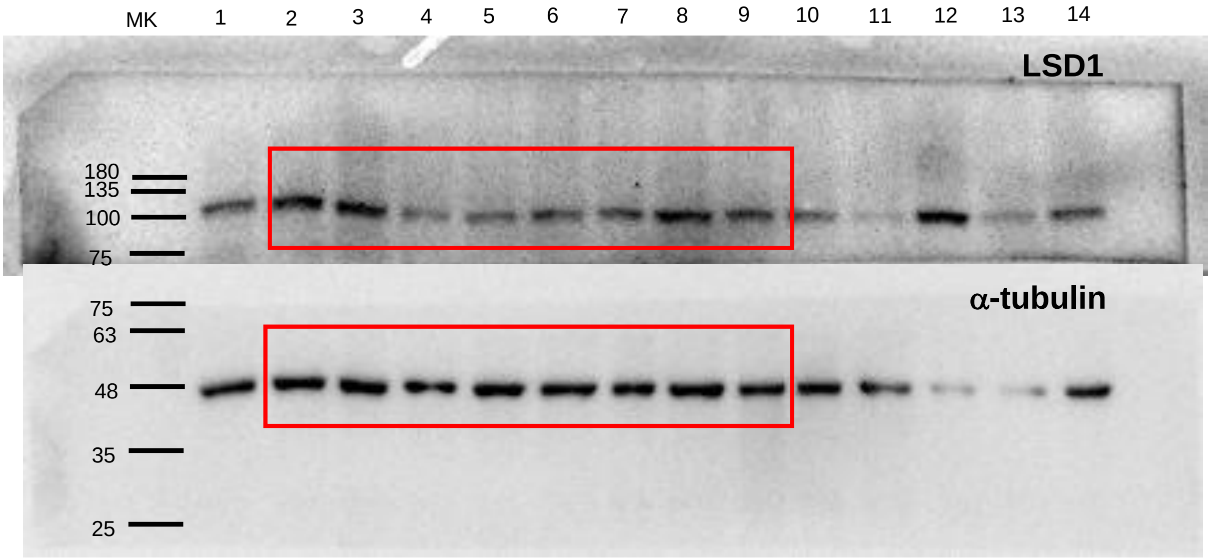

Lanes: MK) molecular weight marker (kDa), 1) brain frontal cortex, 2) veh1, 3) veh2, 4) sch1, 5) sch2, 6) coc1, 7) coc2, 8) sch-coc1, 9) sch-coc2, 10) veh3, 11) sch3, 12) coc3, 13) sch-coc3, 14) naive

KMT1C/G9a

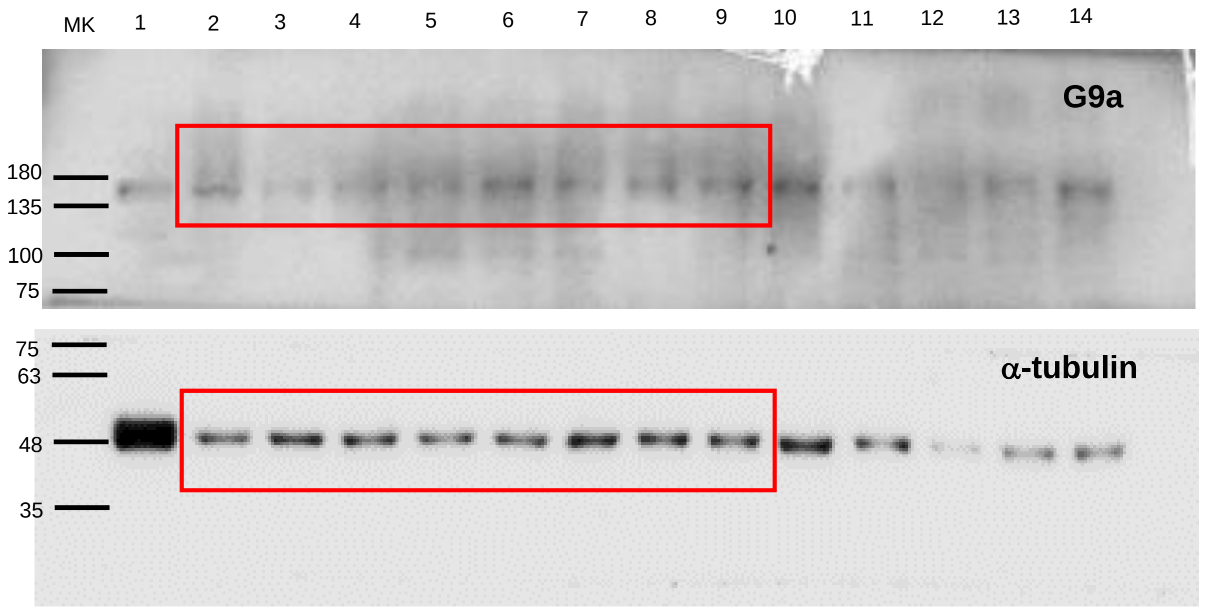

Lanes: MK) molecular weight marker (kDa), 1) brain frontal cortex, 2) veh1, 3) veh2, 4) sch1, 5) sch2, 6) coc1, 7) coc2, 8) sch-coc1, 9) sch-coc2, 10) coc3, 11) sch3, 12) coc4, 13) sch-coc4, 14) veh3
